# Supplementary material for: Psychometric Properties of Fears of Cancer Recurrence Scales in Turkish Hematologic Cancer Patients
Source: Medicina (Kaunas). 2025 Sep 9;61(9):1628. doi: 10.3390/medicina61091628 (PMC12471791; doi:10.3390/medicina61091628)
Supplement: Supplementary file 1 [file medicina-61-01628-s001.zip › medicina-3832925-supplementary.pdf]

Table S1. Goodness-of-Fit Indices for Alternative Models of FCR-7, FCR-4, and FCR-6 for employed participants.

**Models**	χ^2	*df*	*p*	**CFI**	**TLI**	**RMSEA**	**RMSEA *p***	**LB**	**UB**	**SRMR**
**FCR7**										
Model 1	23.969	14	0.001***	0.948	0.922	0.105	0.110	0.014	0.174	0.035
Model 2	18.521	13	0.139	0.971	0.954	0.081	0.252	0.000	0.158	0.027
Model 3	22.649	13	0.046*	0.950	0.919	0.107	0.107	0.014	0.179	0.034
Model 4	22.430	13	0.049*	0.951	0.921	0.106	0.113	0.007	0.178	0.036
Model 5	21.110	13	0.071	0.958	0.932	0.098	0.150	0.000	0.171	0.031
**FCR4**										
Model 1	4.821	2	0.090	0.982	0.946	0.147	0.125	0.000	0.321	0.025
Model 2	.241	1	0.623	1.00	1.029	0.000	0.651	0.000	0.259	0.003
**FCR6**										
Model 1	20.609	9	0.015	0.938	0.897	0.141	0.037*	0.059	0.222	0.037
Model 2	12.214	8	0.142	0.974	0.952	0.090	0.231	0.000	0.185	0.231
Model 3	15.928	8	0.043*	0.952	0.909	0.123	0.088	0.021	0.212	0.030
Model 4	14.600	8	0.067	0.960	0.924	0.113	0.127	0.000	0.203	0.030
Model 5	16.256	8	0.039*	0.950	0.905	0.126	0.081	0.027	0.214	0.030

Note. FCR7 = Fears of Cancer Recurrence-7 Item Version; FCR4 = Fears of Cancer Recurrence -4 Item Version; FCR6 = Fears of Cancer Recurrence-6 Item Version., $p < 0.05$*, $p < 0.001$***.

Table S2. Goodness-of-Fit Indices for Alternative Models of FCR-7, FCR-4, and FCR-6 for unemployed participants.

**Models**	χ^2	*df*	*p*	**CFI**	**TLI**	**RMSEA**	**RMSEA *p***	**LB**	**UB**	**SRMR**
**FCR7**										
Model 1	34.923	14	.002**	0.958	0.937	0.102	0.024*	0.060	0.145	0.040
Model 2	25.107	13	.022*	0.976	0.961	0.080	0.135	0.030	0.127	0.034
Model 3	30.824	13	.004**	0.964	0.942	0.098	0.041*	0.053	0.143	0.037
Model 4	29.686	13	.005**	0.966	0.946	0.094	0.052	0.049	0.140	0.041
Model 5	22.379	13	.050*	0.981	0.969	0.071	0.221	0.002	0.119	0.032
**FCR4**										
Model 1	4.344	2	0.114	0.995	0.985	0.090	0.204	0.000	0.209	0.012
Model 2	0.634	1	0.426	1.00	1.005	0.000	0.504	0.000	0.203	0.005
**FCR6**										
Model 1	17.923	9	0.036*	0.979	0.965	0.083	0.149	0.020	0.139	0.032
Model 2	11.419	8	0.179	0.992	0.985	0.054	0.399	0.000	0.120	0.026
Model 3	15.714	8	0.047*	0.982	0.966	0.082	0.168	0.010	0.141	0.030
Model 4	11.350	8	0.183	0.992	0.985	0.054	0.404	0.000	0.120	0.030
Model 5	17.374	8	0.026*	0.978	0.959	0.090	0.114	0.029	0.149	0.032

Note. FCR7 = Fears of Cancer Recurrence-7 Item Version; FCR4 = Fears of Cancer Recurrence -4 Item Version; FCR6 = Fears of Cancer Recurrence-6 Item Version., $p < 0.05$*, $p < 0.01$**.

Table S3. Goodness-of-Fit Indices for Alternative Models of FCR-7, FCR-4, and FCR-6 for participants with multiple myeloma.

Models	χ^2	*df*	*p*	CFI	TLI	RMSEA	RMSEA *p*	LB	UB	SRMR
**FCR7**										
Model 1	18.605	14	0.181	0.967	0.950	0.080	0.283	0.000	0.167	0.053
Model 2	17.751	13	0.167	0.966	0.945	0.085	0.263	0.000	0.174	0.051
Model 3	17.836	13	0.164	0.965	0.944	0.085	0.259	0.000	0.174	0.052
Model 4	17.923	13	0.161	0.965	0.943	0.086	0.254	0.000	0.175	0.052
Model 5	13.637	13	0.400	0.995	0.993	0.031	0.521	0.000	0.144	0.046
**FCR4**										
Model 1	1.655	2	0.437	1.00	1.008	0.000	0.482	0.000	0.262	0.013
Model 2	0.962	1	0.327	1.00	1.002	0.000	0.357	0.000	0.357	0.010
**FCR6**										
Model 1	8.805	9	0.456	1.00	1.003	0.000	0.555	0.000	0.155	0.041
Model 2	8.181	8	0.416	0.998	0.997	0.021	0.511	0.000	0.166	0.040
Model 3	3.669	8	0.886	1.00	1.068	0.000	0.919	0.000	0.078	0.020
Model 4	8.512	8	0.385	0.996	0.992	0.035	0.480	0.000	0.170	0.041
Model 5	8.436	8	0.392	0.996	0.993	0.033	0.487	0.000	0.170	0.041

Note. FCR7 = Fears of Cancer Recurrence-7 Item Version; FCR4 = Fears of Cancer Recurrence -4 Item Version; FCR6 = Fears of Cancer Recurrence-6 Item Version.

Table S4. Goodness-of-Fit Indices for Alternative Models of FCR-7, FCR-4, and FCR-6 for participants with lymphoma.

Models	χ^2	*df*	*p*	CFI	TLI	RMSEA	RMSEA *p*	LB	UB	SRMR
**FCR7**										
Model 1	35.061	14	0.001**	0.950	0.926	0.108	0.019*	0.064	0.153	0.036
Model 2	20.632	13	0.081	0.982	0.971	0.067	0.269	0.000	0.120	0.028
Model 3	32.738	13	0.002**	0.952	0.925	0.108	0.021*	0.063	0.156	0.034
Model 4	28.454	13	0.008**	0.964	0.941	0.096	0.058	0.047	0.144	0.036
Model 5	30.148	13	0.005**	0.960	0.935	0.101	0.040*	0.054	0.149	0.032
**FCR4**										
Model 1	9.567	2	0.008**	0.980	0.939	0.171	0.024*	0.074	0.287	0.020
Model 2	0.562	1	0.453	1.00	1.007	0.000	0.522	0.000	0.210	0.004
**FCR6**										
Model 1	25.161	9	0.003*	0.957	0.928	0.118	0.022*	0.065	0.174	0.033
Model 2	13.510	8	0.096	0.985	0.972	0.073	0.249	0.000	0.138	0.025
Model 3	23.899	8	0.002**	0.957	0.920	0.124	0.018*	0.069	0.183	0.033
Model 4	18.022	8	0.021*	0.973	0.950	0.099	0.087	0.036	0.160	0.031
Model 5	24.367	8	0.002**	0.956	0.918	0.126	0.015*	0.071	0.185	0.033

Note. FCR7 = Fears of Cancer Recurrence-7 Item Version; FCR4 = Fears of Cancer Recurrence -4 Item Version; FCR6 = Fears of Cancer Recurrence-6 Item Version, $p < 0.05$*, $p < 0.01$**.

Table S5. Results of Confirmatory Factor Analysis for FCR-7, FCR-4, and FCR-6.

**Item number**	***λ***	**SE**	***R*²**
**FCR7**			
Item1	0.83	0.03	0.69
Item2	0.88	0.03	0.77
Item3	0.90	0.02	0.81
Item4	0.87	0.03	0.75
Item5	0.83	0.04	0.69
Item6	0.51	0.07	0.26
Item7	0.57	0.05	0.32
**FCR4**			
Item1	0.84	0.03	0.70
Item2	0.90	0.03	0.80
Item3	0.92	0.03	0.85
Item4	0.83	0.03	0.69
**FCR6**			
Item1	0.83	0.03	0.69
Item2	0.88	0.03	0.77
Item3	0.90	0.02	0.81
Item4	0.87	0.03	0.75
Item5	0.83	0.04	0.68
Item6	0.57	0.05	0.32

Note. FCR7 = Fears of Cancer Recurrence-7 Item Version; FCR4 = Fears of Cancer Recurrence -4 Item Version; FCR6 = Fears of Cancer Recurrence-6 Item Version. λ = standardized item factor loadings, SE = Standard error, R^2= The proportion of the variance for a variable that is explained by the latent factor in the model. All item factor loadings were significant at least at the $p < 0.001$ level.
